# Functional alignment in robotic total knee arthroplasty achieves comparable outcomes in varus and valgus knees despite distinct intraoperative strategies: Analysis of 355 consecutive cases

**DOI:** 10.1002/ksa.12764

**Published:** 2025-07-07

**Authors:** Christos Koutserimpas, Clemente Caria, Pietro Gregori, Luca Andriollo, Elvire Servien, Cécile Batailler, Sébastien Lustig

**Affiliations:** ^1^ School of Rehabilitation Health Sciences University of Patras Patras Greece; ^2^ Orthopaedics Surgery and Sports Medicine Department, FIFA Medical Center of Excellence, Croix‐Rousse Hospital Lyon University Hospital Lyon France; ^3^ LIBM‐EA 7424, Interuniversity Laboratory of Biology of Mobility Claude Bernard Lyon 1 University Lyon France; ^4^ Univ Lyon, Claude Bernard Lyon 1 University, IFSTTAR, LBMC UMR_T9406 Lyon France

**Keywords:** coronal alignment, functional alignment, functional knee positioning, robotic knee

## Abstract

**Purpose:**

Functional alignment (FA) optimises implant positioning based on patient‐specific anatomy, but data on intraoperative adjustments in varus and valgus knees remain limited. This study evaluates the impact of preoperative coronal alignment on implant positioning, bone resections, functional outcomes, and revision rates in robotic‐assisted total knee arthroplasty (TKA) under FA principles.

**Methods:**

A retrospective analysis of 355 robotic‐assisted TKAs performed with FA was conducted. Patients were classified as varus (*n* = 294, HKA < 180°) or valgus (*n* = 61, HKA ≥ 180°). Intraoperative implant positioning, bone resections, and alignment parameters were recorded. Functional outcomes were assessed using the Knee Society Scores, Forgotten Joint Score, and Kujala score. Implant survivorship was analysed using the Kaplan–Meier method.

**Results:**

Varus knees required greater tibial varus positioning (3.5° varus [interquartile range [IQR] 2–5] vs. 1° varus [IQR 0–2.5], *p* < 0.0001), while valgus knees required increased femoral valgus positioning (1.5° [IQR 0.38–2.5] vs. 0.7° [IQR −0.5 to 1.83], *p* = 0.0004). Bone resections also differed significantly between groups. Specifically, the valgus group had lower lateral tibial (*p* = 0.0001), distal lateral (*p* < 0.0001), and posterior lateral femoral resections (*p* < 0.0001), but higher distal medial femoral resections (*p* = 0.04). Postoperative functional outcomes were comparable across groups. Survival rates were 98.64% (varus) and 98.36% (valgus) (*p* = 0.86), with a hazard ratio of 1.23 (95% CI: 0.12–12.57) for valgus knees.

**Conclusion:**

This study systematically evaluates intraoperative modifications in FA‐based robotic TKA for varus and valgus knees. Despite distinct balancing strategies, both groups achieved comparable outcomes and implant survivorship.

**Level of Evidence:**

Level III.

**Abbreviations**
BMI: body mass index
CI: confidence interval
CT: computed tomography
FA: functional alignment
FJS‐12: Forgotten Joint Score
HKA: hip‐knee‐ankle angle
IQR: interquartile range
KSS: Knee Society Score
LDFA: Lateral distal femoral angle
MA: mechanical alignment
MPTA: medial proximal tibial angle
ROM: range of motion
TEA: trans‐epicondylar axis
TKA: total knee arthroplasty

## INTRODUCTION

Coronal deformities in total knee arthroplasty (TKA) are traditionally categorised as varus or valgus based on the hip‐knee‐ankle (HKA) angle, with varus knees commonly associated with medial compartment wear, collateral ligament contractures, and increased tibial varus inclination, whereas valgus knees often present with lateral compartment degeneration, femoral valgus orientation, and ligamentous laxity or insufficiency [5, 9, 15, 40, 51, 52]. It should be noted that this binary classification, although broadly used in clinical practice, has been increasingly questioned by newer concepts such as functional knee phenotypes and the Coronal Plane Alignment of the Knee (CPAK) classification [11, 24, 42]. Traditional mechanical alignment (MA) in TKA restores a neutral limb axis by positioning components perpendicular to the mechanical axes, often overlooking native anatomical and soft tissue variations [8]. his approach may disrupt joint biomechanics and lead to suboptimal outcomes, prompting growing interest in alignment strategies that respect individual morphology [6, 21].

To address the limitations of mechanical alignment, functional alignment (FA) or functional knee positioning has emerged as an alternative approach that seeks to restore the knee's native biomechanics while optimising implant positioning based on patient‐specific anatomical characteristics [32, 43, 44]. Unlike mechanical alignment, which aims for a uniform neutral axis, FA considers factors such as preoperative limb alignment, soft tissue balance, and the anterior compartment to achieve a more individualised reconstruction [3, 12, 19, 31, 37, 38, 39, 46, 48]. FA is inherently based on robotic technology, which has been proven safe and reliable and it enables controlled coronal, sagittal, and rotational adjustments tailored to each patient's unique morphology [1, 2, 4, 16, 17, 18, 33, 45]. However, as FA is a relatively new concept, there is a lack of data on how different factors influence clinical outcomes, implant positioning, and revision rates. In particular, it remains unclear how implant adjustments and bone resections differ between varus and valgus knees when balancing the joint under FA principles.

The aim of this retrospective comparative study was to evaluate the impact of preoperative coronal alignment (in terms of the broadly used in clinical practice distinction between varus and valgus) on implant positioning, bone resections, functional outcomes, and revision rates in patients undergoing robotic‐assisted TKA using the image‐based robotic system guided by FA principles, with a minimum follow‐up of 2 years.

## METHODS

This retrospective comparative study analysed a prospectively maintained database of patients who underwent primary robotic‐assisted TKA using the FA principles without soft tissue releases [19, 48, 49]. The study period was from March 2021 to March 2023. Patients were categorised into two groups based on their preoperative HKA measured from full‐length standing radiographs. Those with an HKA < 180° were classified as having a varus deformity, while those with an HKA ≥ 180° were categorised as having a valgus deformity. All procedures were performed using the Mako, Stryker (Mako Surgical Corp., Fort Lauderdale, FL, USA) robotic system, while all patients received a cruciate‐substituting or posterior‐stabilised fixed‐bearing implant (Triathlon, Stryker, MI, USA). The minimum follow‐up period for all patients was 24 months to ensure sufficient postoperative assessment of clinical and radiographic parameters.

Patients were excluded if they had undergone robotic TKA using mechanical alignment principles due to pre‐existing soft tissue deficiencies (*n* = 112). Additional exclusions included cases with incomplete preoperative imaging and/or intraoperative robotic data (*n* = 22), patients lost to follow‐up before 24 months (*n* = 18), and those who received constrained implants (*n* = 4).

A total of 355 patients were included in the study, with a median age of 70 years (interquartile range [IQR] 64–74) and a median body mass index (BMI) of 27.78 kg/m² (IQR 24.91–31.79). The cohort consisted of 205 females (57.75%), and the mean follow‐up duration was 33.14 months (SD = 5.86). A total of 294 patients were included in the varus and 61 in the valgus group.

Preoperative and postoperative radiographic evaluation included the HKA, lateral distal femoral angle (LDFA), and medial proximal tibial angle (MPTA), all measured from full‐length standing radiographs, while the tibial posterior slope was assessed using standardised lateral knee radiographs. Two independent investigators (fellowship‐trained orthopaedic surgeons) conducted all measurements using the tools provided within the picture archiving and communication system, recording values to the nearest 0.1°. Inter‐observer reliability was evaluated through intraclass correlation coefficients (ICC), demonstrating excellent agreement: ICC = 0.92 for HKA, 0.93 for LDFA, and 0.91 for MPTA.

Intraoperatively, coronal alignment was also assessed through the robotic system, which provided real‐time varus/valgus alignment measurements based on the preoperative computed tomography (CT) scan.

**Figure 1:**
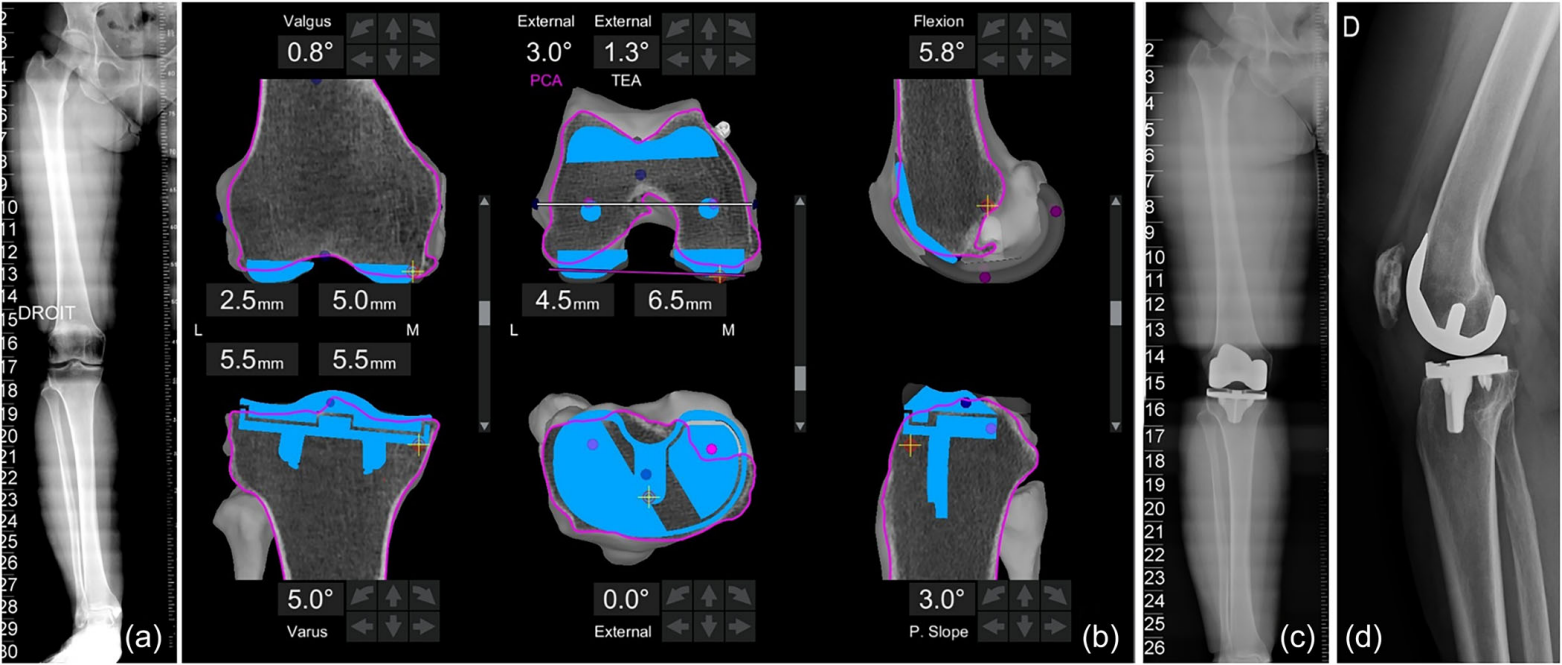
An example of a varus case undergoing robotic total knee arthroplasty with the functional alignment principles. The case refers to a 70‐year‐old female with body mass index = 37.22 kg/m^2^. (a) The long axis preoperative X‐ray showed hip‐knee‐angle (HKA) = 174°. (b) The final planning with the image‐based platform: the femoral implant is placed in 0.8° of valgus and 5.8° of flexion (both in reference to the mechanical axis of the femur), as well as 1.3° external rotation (in reference to the trans‐epicondylar axis). The tibia is placed in 5° of varus and 3° posterior slope (both in reference to the mechanical axis of the tibia), as well as in 0 of external rotation (in reference to the Akagi line). (c) The postoperative long axis anteroposterior x‐ray shows HKA = 178°. (d) The lateral postoperative X‐ray.

**Figure 2:**
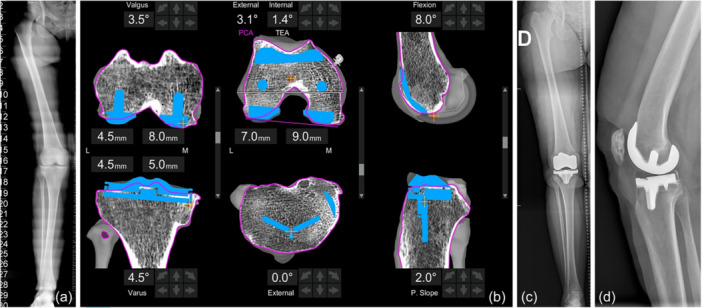
An example of a valgus case undergoing robotic total knee arthroplasty with the functional alignment principles. The case refers to a 68‐year‐old female with body mass index = 26.6 kg/m^2^. (a) the long axis preoperative X‐ray showed hip‐knee‐angle (HKA) = 190°. (b) The final planning with the image‐based platform: the femoral implant is placed in 3.5° of valgus and 8° of flexion (both in reference to the mechanical axis of the femur), as well as 1.4° internal rotation (in reference to the trans‐epicondylar axis). The tibia is placed in 4.5° of varus and 2° posterior slope (both in reference to the mechanical axis of the tibia), as well as in 0 of external rotation (in reference to the Akagi line). (c) The postoperative long axis anteroposterior x‐ray shows HKA = 182°. (d) The lateral postoperative X‐ray.

Implant positioning was analysed intraoperatively through the robotic system, which provided real‐time data on femoral and tibial coronal alignment, femoral flexion, and tibial posterior slope, referenced to the mechanical axis of the femur and tibia, respectively. Femoral component rotation was determined relative to the surgical trans‐epicondylar axis (TEA), while tibial component rotation was referenced to the Akagi line (Figures 1 and 2).

The thickness of bone resections was recorded for both the femur and tibia, including medial and lateral tibial cuts, distal medial and distal lateral femoral cuts, as well as posterior medial and posterior lateral femoral cuts. All measurements were obtained intraoperatively through the robotic system's tracking and guidance software.

Preoperative and postoperative functional outcomes were assessed using the Knee Society Scores (KSS) for knee function and overall performance. Range of motion (ROM) was evaluated based on active knee flexion, while the Forgotten Joint Score (FJS‐12) and Kujala score were recorded at the final follow‐up to assess joint awareness and anterior knee function, respectively.

Revisions were categorised as all‐cause and aseptic, with all‐cause revisions including any reoperations requiring component exchange, while aseptic revisions specifically excluded infections. Survival analysis was conducted using the Kaplan–Meier method, comparing implant survivorship between the varus and valgus groups.

### Statistical analyses

Distribution was evaluated with the Kolmogorov–Smirnov test. Independent t‐test or Mann–Whitney was used to compare groups based on the presence or absence of normality. The Chi‐square test for categorical data, including revision rates. Statistical significance was set at *p* < 0.05. Implant survival was analysed using the Kaplan–Meier method, and all statistical analyses were performed using MedCalc software (version 22.021). A power analysis was not performed, as this retrospective study was based on an all‐comers design including all consecutive eligible cases during the study period.

## RESULTS

Preoperative evaluation confirmed significant differences in coronal alignment between the groups, with varus knees showing lower HKA and MPTA, and valgus knees presenting higher LDFA values (*p* < 0.0001 for HKA and MPTA and 0.004 for LDFA; Table 1). Intraoperative robotic assessment also revealed a greater varus alignment in the varus group (*p* < 0.0001). Additionally, BMI was significantly lower in the valgus group (*p* = 0.01), while the distribution of insert types was similar between groups.

**Table 1:** Preoperative comparative data between the two groups (varus and valgus).

		Varus group (*N* = 294)	Valgus group (*N* = 61)	*p* value
Demographics	Age (years)	70 (IQR 64–74)	70 (IQR 63–74)	0.84
	Female gender	58.16%	55.74%	0.73
	BMI (kg/m^2^)	28.13 (IQR 25.39–32)	25.95 (IQR 23.17–30.35)	**0.01**
Type of insert	CS	35.03%	31.15%	0.56
Preoperative clinical evaluation	KSS‐knee	65 (IQR 57–75)	65.5 (IQR 56–71)	0.81
	KSS‐function	70 (IQR 60–80)	70 (IQR 60–80)	0.79
	Active knee flexion (clinical evaluation)	120° (IQR 110–130)	120° (IQR 110–130)	0.34
Preoperative radiological evaluation	HKA	173° (IQR 170–177)	186° (IQR 182.75–188)	**<0.0001**
	LDFA	91° (IQR 90–92)	93° (IQR 89.75–95)	**0.004**
	MPTA	86° (IQR 84–88)	90° (IQR 88–92)	**<0.0001**
	Tibial posterior slope	7° (IQR 5–9)	7° (IQR 4–9)	0.76
Intraoperative assessment with the robotic system	Alignment (varus)	7° (IQR 4–9)	−1° (IQR −3 to 2)	**<0.0001**

*Note*: Statistically significant differences are indicated in bold.

Abbreviations: BMI, body mass index; HKA, Hip‐Knee angle; IQR, interquartile range; KSS, Knee Society Score; LDFA, lateral distal femoral angle; MPTA, medial proximal tibial angle; *N*, number.

Intraoperative analysis of implant positioning showed that tibial coronal alignment was 3.5° varus (IQR 2–5) in the varus group and 1° varus (IQR 0–2.5) in the valgus group (*p* < 0.0001), while the median tibial posterior slope was 1° (IQR 0–1) and 0° (IQR 0–1) (*p* = 0.01) in the varus and valgus group, respectively. The femoral component valgus alignment measured 0.7° (IQR −0.5 to 1.83) in the varus group and 1.5° (IQR 0.38–2.5) in the valgus group (*p* = 0.0004). The valgus group also had a greater femoral component flexion angle (9° [IQR 7.33–10) vs. 7° [IQR 5–9], *p* < 0.0001). No significant differences were observed in femoral component rotation (Table 2).

**Table 2:** Femoral and tibial implant positioning during the robotic‐assisted total knee arthroplasty under the functional alignment principles.

		Varus group (*N* = 294)	Valgus group (*N* = 61)	*p*‐value
Tibial implant positioning (degrees with reference to the tibial mechanical axis)	Varus	3.5 (IQR 2–5)	1 (IQR 0–2.5)	**<0.0001**
	Posterior slope	1 (IQR 0–1)	0 (IQR 0–1)	**0.01**
Femoral implant positioning (degrees with reference to the femoral mechanical axis)	External rotation (reference TEA)	0.1 (IQR −1.3 to 1.3)	0.5 (IQR −0.48 to 1.38)	0.28
	Valgus	0.7 (IQR −0.5 to 1.83)	1.5 (IQR 0.38–2.5)	**0.0004**
	Flexion	7 (IQR 5–9)	9 (IQR 7.33–10)	**<0.0001**

*Note*: Statistically significant differences are indicated in bold.

Abbreviations: IQR, interquartile range; TEA, trans‐epicondylar axis.

Analysis of bone resection thickness showed that the lateral tibial cut was lower in the valgus group compared to the varus group (7 mm (IQR 5–8) vs. 8 mm (IQR 7–8.63), *p* = 0.0001). Regarding femoral resections, the distal medial cut was higher in the valgus group (9 mm [IQR 9–10] vs. 9 mm [IQR 8–9.5], *p* = 0.04), while the distal lateral cut was lower (6.5 mm [IQR 5.5–7] vs. 8.5 mm [IQR 7–9], *p* < 0.0001). The posterior lateral cut was lower in the valgus group (7 mm [IQR 6–8] vs. 8.5 mm [IQR 7.5–9], *p* < 0.0001). No significant differences were observed in medial tibial (*p* = 0.91) and the femoral posterior medial cut thickness (*p* = 0.13) (Table 3).

**Table 3:** Evaluation of the thickness of the cuts between the two groups (varus and valgus).

		Varus group (*N* = 294)	Valgus group (*N* = 61)	*p* value
Tibial cuts (mm)	Medial	7.5 (IQR 6.38–8)	7.5 (IQR 6.5–8)	0.74
	Lateral	8 (IQR 7–8.63)	7 (IQR 5–8)	**0.0001**
Femoral cuts (mm)	Distal medial	9 (IQR 8–9.5)	9 (IQR 9–10)	**0.04**
	Distal lateral	8.5 (IQR 7–9)	6.5 (IQR 5.5–7)	**<0.0001**
	Posterior medial	9 (IQR 8.5–9.5)	9 (IQR 9–10)	0.13
	Posterior lateral	8.5 (IQR 7.5–9)	7 (IQR 6–8)	**<0.0001**

*Note*: Statistically significant differences are indicated in bold.

Abbreviation: IQR, interquartile range.

Postoperative analysis confirmed residual differences in HKA and robotic system alignment between the varus and valgus groups (*p* < 0.0001). However, no significant differences were observed in clinical outcomes at final follow‐up, including KSS (knee and function), FJS‐12, Kujala score, or active knee flexion (Table 4).

**Table 4:** Postoperative evaluation of the clinical, radiographic parameters between the two groups.

		Varus group (*N* = 294)	Valgus group (*N* = 61)	*p* value
Postoperative clinical evaluation	KSS‐knee	95 (IQR 90–100)	94 (IQR 90–100)	0.99
	KSS‐function	90 (IQR 90–100)	94 (IQR 90–100)	0.57
	FJS‐12	86 (IQR 75–92)	90 (IQR 78.5–94)	0.1
	Kujala Score	92 (IQR 84–100)	95 (IQR 90–100)	0.25
	Active knee flexion (degrees)	130 (IQR 120–130)	130 (IQR 120–130)	0.41
Postoperative coronal alignment evaluation	HKA (degrees)	178 (IQR 176–179.5)	181 (IQR 178–182)	**<0.0001**
	LDFA	91 (IQR 89–92)	90 (IQR 88–91)	**0.02**
	MPTA	88 (IQR 86– 89)	89 (IQR 88–91)	**0.001**
Assessment from the robotic system	Postoperative Mako Alignment (varus‐degrees)	3 (IQR 2–5)	0 (IQR −1 to 1)	**<0.0001**

*Note*: Statistically significant differences are indicated in bold.

Abbreviations: FJS‐12, Forgotten Joint Score; HKA, hip‐knee angle; IQR, interquartile range; KSS, Knee Society Score; LDFA, lateral distal femoral angle; MPTA, medial proximal tibial angle; *N*, number.

**Figure 3:**
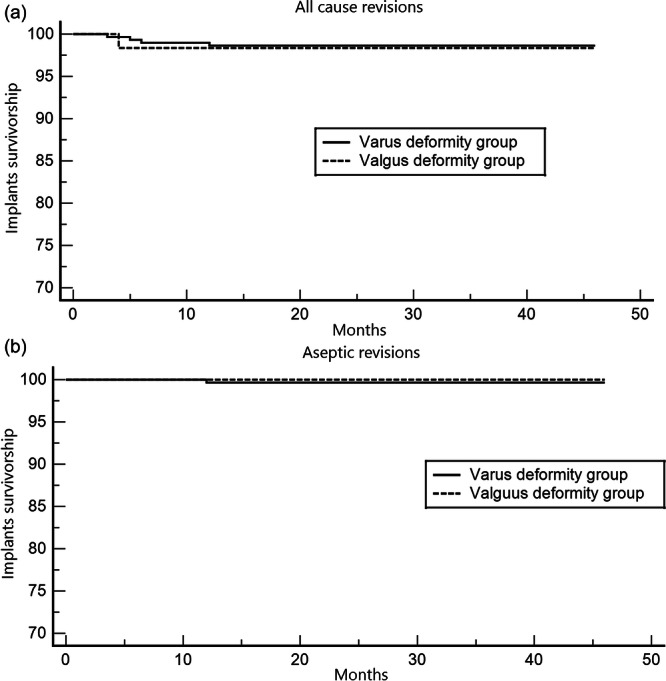
(a) Kaplan–Meier survival analysis for all‐cause revision, comparing the varus and valgus groups. The survival rate was 98.64% in the varus group and 98.36% in the valgus group (*p* = 0.86). The hazard ratio for all‐cause revision was 0.82 (95% CI: 0.08–8.36) for the varus group and 1.23 (95% CI: 0.12–12.57) for the valgus group. (b) Kaplan–Meier survival analysis for aseptic loosening, comparing the varus and valgus groups. The survival rate was 99.66% in the varus group and 100% in the valgus group (*p* = 0.65). CI, confidence interval.

Survival analysis showed no significant difference in all‐cause revision rates between the groups, with survival rates of 98.64% in the varus group and 98.36% in the valgus group (*p* = 0.86). The hazard ratio for all‐cause revision was 0.82 (95% confidence interval [CI]: 0.08–8.36) for the varus group and 1.23 (95% CI: 0.12–12.57) for the valgus group. For aseptic survivorship, one case of aseptic revision was recorded in the varus group, resulting in survival rates of 100% in the valgus group and 99.66% in the varus group (*p* = 0.65) (Figure 3a,b).

## DISCUSSION

This study investigated the influence of preoperative coronal alignment (varus vs. valgus) on implant positioning, bone resections, functional outcomes, and revision rates in robotic‐assisted TKA performed under FA principles. The key findings demonstrated that varus and valgus knees underwent distinct intraoperative adjustments to achieve balance, particularly in terms of implant positioning and bone resection thickness. The valgus group exhibited greater femoral component flexion and valgus alignment, while the varus group had higher tibial coronal varus positioning. Additionally, significant differences were observed in femoral and tibial bone resections, with the valgus group requiring thicker posterior medial and distal medial femoral cuts, while the varus group had greater lateral tibial and posterior lateral femoral resections. These findings suggest that when FA principles are applied, varus knees are primarily adjusted through tibial resections, while valgus knees require more femoral modifications to restore balance. Despite these differences in intraoperative decision‐making, postoperative functional outcomes and implant survivorship were comparable between groups, with no significant differences in KSS, FJS‐12, Kujala score or revision rates.

Preoperative analysis confirmed the expected differences in coronal alignment between groups. The discrepancy between full‐length weight‐bearing radiographs and intraoperative robotic assessment likely reflects methodological differences, with radiographs capturing physiological loading and CT‐based planning conducted in the supine, non‐weight‐bearing position. This divergence, particularly pronounced in valgus knees, aligns with findings by Gregori et al. [20]. Furthermore, the valgus cohort demonstrated higher MPTA and LDFA values, consistent with previous reports highlighting distinct anatomical characteristics of each deformity. Varus knees are commonly associated with medial tibial wear and a tibial plateau inclined in varus, whereas valgus knees tend to present with increased lateral inclination of both the femur and tibia [22, 41, 52]. Additionally, the valgus group in this study had a lower median BMI than the varus group. Although most studies have reported higher BMI in valgus cases, it is evident that patients with valgus deformities often have different anthropometric characteristics and gait adaptations compared to those with varus alignment [13, 47, 50]. However, no significant differences were observed in preoperative KSS, active knee flexion, or posterior tibial slope, indicating that despite differences in coronal alignment, baseline functional status was comparable between groups. Moreover, there was similar distribution of insert types. A recent study revealed comparable functional outcomes and survivorship between cruciate‐substituting and posterior stabilised implants in the setting of robotic TKA with the FA strategy [36].

This study revealed that under FA principles without soft tissue releases, achieving optimal knee balance in varus and valgus deformities necessitated distinct intraoperative adjustments, particularly concerning implant positioning and bone resection thickness. Specifically, valgus knees required greater modifications on the femoral side, including increased femoral component flexion and valgus alignment, as well as thicker distal medial femoral resections. Conversely, varus knees necessitated more substantial adjustments on the tibial side, characterised by higher tibial coronal varus positioning and greater lateral tibial and posterior lateral femoral resections.

It should be noted that in the present study, all cases were operated under the FA with the implants' positioning limits described by Shatrov et al. [48, 49]. In particular, the tibial coronal alignment is set within 6° varus to 2° valgus, and femoral alignment between 6° valgus and 3° varus. These findings highlight a fundamental difference between FA and MA in achieving knee balance. While FA balances the knee through implant positioning and bone resection adjustments, traditional MA relies on soft tissue releases to correct coronal deformities and restore a neutral mechanical axis [8, 29]. Notably, studies using MA have defined strict coronal alignment limits, with optimal tibial component alignment typically set between 2° and 4° varus, and femoral alignment between 0° and 2° valgus, to ensure even load distribution and implant survivorship [30]. However, these thresholds were established under an MA framework, where ligament releases compensate for rigid component positioning rather than tailoring the implant alignment to native knee morphology, while a recent study showed that expanding the tibial varus positioning up to 6° in FA strategy seems to be safe [7, 34]. In contrast, FA seeks to preserve soft tissue integrity by adjusting implant positioning and bone cuts within controlled limits [19, 48]. This approach aligns with recent findings that suggest minor modifications in implant positioning can eliminate the need for medial or lateral soft tissue releases altogether [53]. These insights further reinforce the rationale behind FA. Rather than imposing a universal alignment target, the knee is balanced by respecting the native soft tissue envelope, which serves as the “DNA” of the knee, guiding the necessary adjustments for each specific patient [20, 35]. By adapting bone resections and implant positioning to the patient's intrinsic ligamentous structure, FA ensures that alignment is achieved in harmony with the existing soft tissue constraints rather than altering them through extensive releases.

The present study showed that both varus and valgus knee deformities, when addressed using FA principles, resulted in comparable and favourable postoperative functional outcomes. Notably, recent studies have highlighted that FA may offer superior outcomes in valgus knees compared to kinematic alignment (KA). For instance, a study by Clark et al. demonstrated that FA more consistently achieves a balanced TKA than either mechanical alignment or KA prior to soft tissue releases [14]. Additionally, Kafelov et al. reported that FA principles in image‐based robotic‐assisted TKA led to higher Forgotten Joint Scores at one year postoperatively compared to conventional TKA with restricted KA [27]. Furthermore, a study by Gregori et al. reported that FA in robotic‐assisted TKA for valgus deformities achieves safe coronal alignment and excellent short‐term outcomes [20]. This suggests that FA's individualised approach, which considers the patient's unique anatomy and soft tissue envelope, may be particularly beneficial in complex deformities such as valgus knees.

Furthermore, the current study showed similar favourable survival rates between the two groups. This finding aligns with previous research evaluating implant survival in different preoperative alignment groups. The study by Kahlenberg et al. reported no significant difference in revision rates between varus and valgus knees at mid‐term follow‐up, reinforcing the notion that modern surgical techniques can optimise outcomes regardless of preoperative deformity [28]. Similarly, Schexnayder et al. found that valgus and varus knees do not exhibit different survival risks post‐TKA, provided that proper alignment and soft tissue balancing are achieved intraoperatively [47].

This study has some limitations that should be acknowledged. It is a single‐centre retrospective study, which inherently introduces potential biases and limits generalisability. While the use of a single surgical technique and robotic system ensures methodological consistency, a multicenter study would better capture variations in surgical execution and patient populations. Additionally, the classification of knees into varus and valgus groups was based solely on the HKA angle, using a threshold of 180°, which, while consistent with prior studies, may oversimplify the coronal alignment spectrum. Most patients in each group had clear deformity patterns (median HKA of 173° for varus and 186° for valgus), yet the lack of a defined neutral group and the absence of joint line convergence angle consideration could obscure more subtle differences in alignment phenotypes. Importantly, functional knee phenotyping and the CPAK classification were not applied in this analysis. While this was intentional due to the study's focus on current clinical decision‐making based on varus and valgus distinctions, we recognise that these more refined classification systems could offer valuable insights into native morphology and alignment‐specific outcomes [10, 23, 25, 26, 42]. Their integration could better stratify patient subtypes and further optimise the functional alignment workflow. Finally, although several statistically significant differences in implant positioning and bone resections were observed between groups, the absolute differences were small, and their clinical relevance remains uncertain. future studies should incorporate CPAK and FKP classifications to validate and personalise intraoperative decision‐making in FA‐based TKA and to move beyond conventional alignment labels. Despite these limitations, this study is the first to evaluate the specific intraoperative adjustments required to achieve balance in varus versus valgus knees under FA principles. It provides important initial data on whether femoral or tibial modifications are more critical in achieving knee balance in different preoperative coronal morphologies.

## CONCLUSIONS

This study provides novel insights into how preoperative coronal alignment (varus vs. valgus) influences implant positioning, bone resections, and intraoperative adjustments in robotic‐assisted TKA performed under FA principles. The findings demonstrate that achieving knee balance in FA differs between varus and valgus knees, with varus cases requiring greater tibial modifications, while valgus cases undergo more extensive femoral adjustments. Despite these intraoperative differences, both groups achieved comparable postoperative functional outcomes and implant survivorship, reinforcing the safety and reliability of FA in addressing different preoperative deformities.

## AUTHOR CONTRIBUTIONS

*Conceptualisation*: Christos Koutserimpas, Clemente Caria, Cécile Batailler and Sébastien Lustig. *Methodology*: Clemente Caria, Elvire Servien, Cécile Batailler and Sébastien Lustig. *Formal analysis and investigation*: Christos Koutserimpas, Clemente Caria, Pietro Gregori and Luca Andriollo. *Writing–original draft preparation*: Christos Koutserimpas, Clemente Caria, Pietro Gregori and Luca Andriollo. *Writing–review and editing*: Elvire Servien, Cécile Batailler and Sébastien Lustig. *Supervision*: Cécile Batailler and Sébastien Lustig.

## CONFLICT OF INTEREST STATEMENT

Christos Koutserimpas, Clemente Caria, Pietro Gregori and Luca Andriollo have nothing to declare. Elvire Servien: Consultant for Smith and Nephew. Cécile Batailler: Consultant for Smith and Nephew and Stryker. Sébastien Lustig: Consultant for Heraeus, Stryker, Depuy Synthes, Smith and Nephew. Institutional research support to Lepine and Amplitude.

## ETHICS STATEMENT

All procedures were performed in accordance with the ethical standards of the institutional and/or national research committee, the 1964 Helsinki declaration and its later amendments, or comparable ethical standards. Data collection and analysis were carried out in accordance with MR004 Reference Methodology from the Commission Nationale de l'Informatique et des Libertés (Ref. 2229975V0). As per institutional standards (Croix Rousse University Hospital of Lyon, France), formal patient consent is not required for this type of study.

## Data Availability

The data that support the findings of this study are available from the corresponding author, upon reasonable request.
